# Hydrothermal Synthesis of a Technical Lignin-Based Nanotube for the Efficient and Selective Removal of Cr(VI) from Aqueous Solution

**DOI:** 10.3390/molecules28155789

**Published:** 2023-07-31

**Authors:** Qiongyao Wang, Yongchang Sun, Mingge Hao, Fangxin Yu, Juanni He

**Affiliations:** 1 Key Laboratory of Subsurface Hydrology and Ecological Effects in Arid Region, Ministry of Education, School of Water and Environment, Chang’an University, Xi’an 710054, China; wqy03218520@163.com (Q.W.); haomingge_813@163.com (M.H.); y526328876@foxmail.com (F.Y.); 2 Huijin Technology Holding Group Corporation Limited, Xi’an 710000, China

**Keywords:** lignin, nanotubes, adsorption, photocatalysis, Cr(VI)

## Abstract

Aminated lignin (AL) was obtained by modifying technical lignin (TL) with the Mannich reaction, and aminated lignin-based titanate nanotubes (AL-TiNTs) were successfully prepared based on the AL by a facile hydrothermal synthesis method. The characterization of AL-TiNTs showed that a Ti–O bond was introduced into the AL, and the layered and nanotubular structure was formed in the fabrication of the nanotubes. Results showed that the specific surface area increased significantly from 5.9 m^2^/g (TL) to 188.51 m^2^/g (AL-TiNTs), indicating the successful modification of TL. The AL-TiNTs quickly adsorbed 86.22% of Cr(VI) in 10 min, with 99.80% removal efficiency after equilibration. Under visible light, AL-TiNTs adsorbed and reduced Cr(VI) in one step, the Cr(III) production rate was 29.76%, and the amount of total chromium (Cr) removal by AL-TiNTs was 90.0 mg/g. AL-TiNTs showed excellent adsorption capacities of Zn^2+^ (63.78 mg/g), Cd^2+^ (59.20 mg/g), and Cu^2+^ (66.35 mg/g). After four cycles, the adsorption capacity of AL-TiNTs still exceeded 40 mg/g. AL-TiNTs showed a high Cr(VI) removal efficiency of 95.86% in simulated wastewater, suggesting a promising practical application in heavy metal removal from wastewater.

## 1. Introduction

Water pollution with heavy metals is commonly more sustainable than organic pollution such as pesticides and dyes, and severe heavy metal pollution could result in devastating effects on humans [1,2]. Particularly, in industrial processes such as metallurgy, galvanization, and tannery, chromium (Cr) is widely used [3,4]. Generally, there are two stable forms of Cr: Cr(III) and Cr(VI) [5]. The high toxicity, carcinogenicity, extreme solubility, and high mobility of Cr(VI), compared to Cr(III), make soil and water pollution treatment extremely difficult [6]. Generally, treatment methods for Cr(VI) pollution include biological reduction, chemical reduction, adsorption, photocatalysis, and electrochemical technologies [7,8]. Among them, adsorption is regarded as a high-performance, low-cost, and green technology in practice [9,10].

Recently, nanotube-based materials have attracted extensive attention as efficient heavy metal adsorption materials [11,12]. For example, a carbon nanotube (CNT)-doped hydrotalcite-hydroxyapatite (HT-HAp) material was prepared to adsorb Cr(VI) from tannery wastewater. Under the action of ion exchange and electrostatic attraction, 76.97% of Cr(VI) was adsorbed by HT-HAp [13]. Guo et al. loaded zirconium oxide (ZrO_2_) onto halloysite nanotubes (ZrO_2_/HNTs) to enhance the average pore size and specific surface area of halloysite nanotubes (HNTs), which resulted in an improved adsorption performance of HNTs on As(III) (36.08 mg/g) [14]. The surface of the synthesized magnetic nanoparticles-phosphate-titanium nanotubes (MNP-PN-TNT) possessed rich hydroxyl groups, which gave MNP-PN-TNT a removal performance of 35 mg/g of Cr(VI) by ion exchange under acidic conditions [15]. Although these nanotube materials exhibited a certain amount of heavy metal adsorption capacity, the high preparation costs significantly hindered their applications [16]. Consequently, it is urgent to develop a cheap and effective method to prepare nanotube materials to remove heavy metals from wastewater [17].

Lignin, as the second most naturally occurring amorphous aromatic molecular polymer in nature after cellulose, is regarded as a key component of the lignocellulosic biomass [18,19]. Technical lignin (TL) is mainly derived from by-products of the paper and pulp industry, and the annual amount of TL produced is estimated to be 225 million tons by 2030 [20]. However, only 5% of TL is utilized at a high-value level, and a large amount of TL is burned as low-value fuel or disposed of as waste [21]. Low utilization is still a challenge for TL [22]. To increase the TL utilization efficiency, chitin was introduced to lignin to prepare chitin/lignin hybrid material for heavy metal removal. It was found that chitin/lignin exhibited superb adsorption capacity of Cu^2+^ (75.70 mg/g), Zn^2+^ (82.41 mg/g), Ni^2+^ (70.41 mg/g), and Pb^2+^ (91.74 mg/g), which was probably attributed to the abundant functional groups of chitin and lignin, such as phenols, hydroxyl, carboxyl, and ethers [23]. In addition, with functional groups and a three-dimensional structure, lignin-based carbon nanotubes (L-CNTs) were prepared by introducing lignin into the carbon nanotubes, which exhibited excellent adsorption performance of L-CNTs for Pb^2+^ (235 mg/g) and the advantages of strong water dispersion and environmental friendliness [24]. The results indicate that lignin is an effective material in heavy metal removal and has a high potential in water pollution treatment.

However, poor water solubility and low surface area remain a challenge for lignin utilization [25,26]. Structural modifications of lignin were reported to enhance the properties of lignin [27]. The main methods for lignin modification are acid hydrolysis, sulfonation, ionic liquid modification, alkylation, steam activation, carboxylation, ball milling, amination, etc. [19,28]. Amination is a prospective method because the amine groups introduced into the lignin ionize and generate a positive charge under acidic conditions. The Mannich reaction for lignin modification is an excellent method because the aminated product is highly dispersed, which improves the lignin adsorption capacity for heavy metals by four to five times [29,30].

**Scheme 1:**
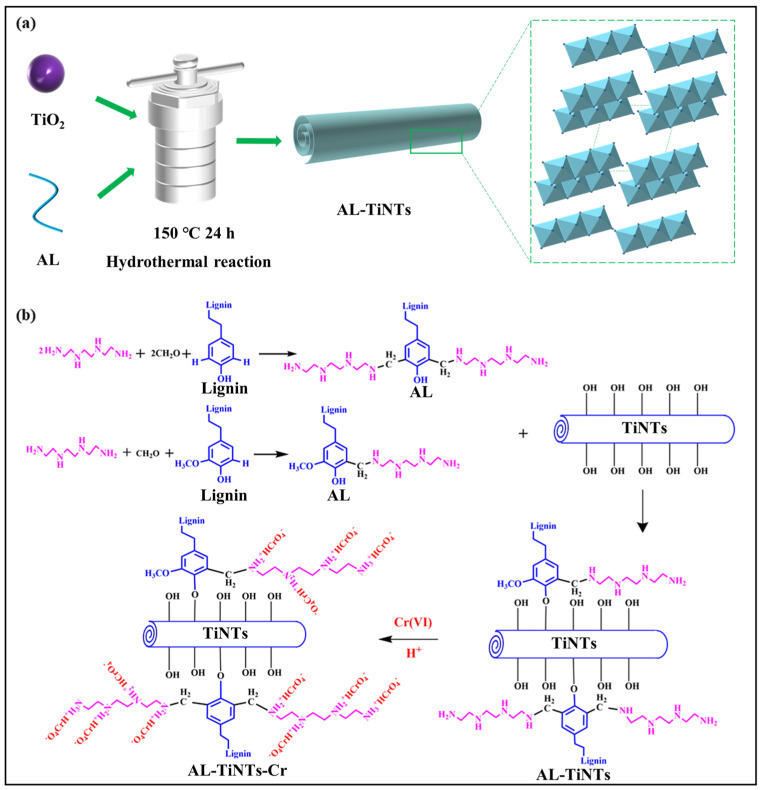
The synthesis process of AL-TiNTs: (**a**) schematic diagram; (**b**) structural diagram.

To our knowledge, titanate nanotubes have been extensively studied as semiconductor materials with high specific surface area and reusable properties [31]. However, studies combining the lignin-rich functional groups with the tubular structure of titanate nanotubes to effectively remove Cr(VI) have not been reported. In this study, TL was modified by activation using the Mannich reaction to improve its disadvantages of easy agglomeration and low adsorption capacity. AL-TiNTs were successfully prepared by a simple hydrothermal method for the first time (Scheme 1). The purpose of this study was to explore (1) the physicochemical properties of AL-TiNTs; (2) the adsorption performance of AL-TiNTs under different conditions; (3) the reduction performance of AL-TiNTs on Cr(VI) in the photocatalytic process; (4) the selectivity of AL-TiNT adsorption in mixed solutions and the wide absorbability on other heavy metal ions; and (5) the removal mechanism of AL-TiNTs for Cr(VI) and the regenerative properties of AL-TiNTs.

## 2. Results and Discussion

### 2.1. Characterization of AL-TiNTs

#### 2.1.1. SEM

**Figure 1:**
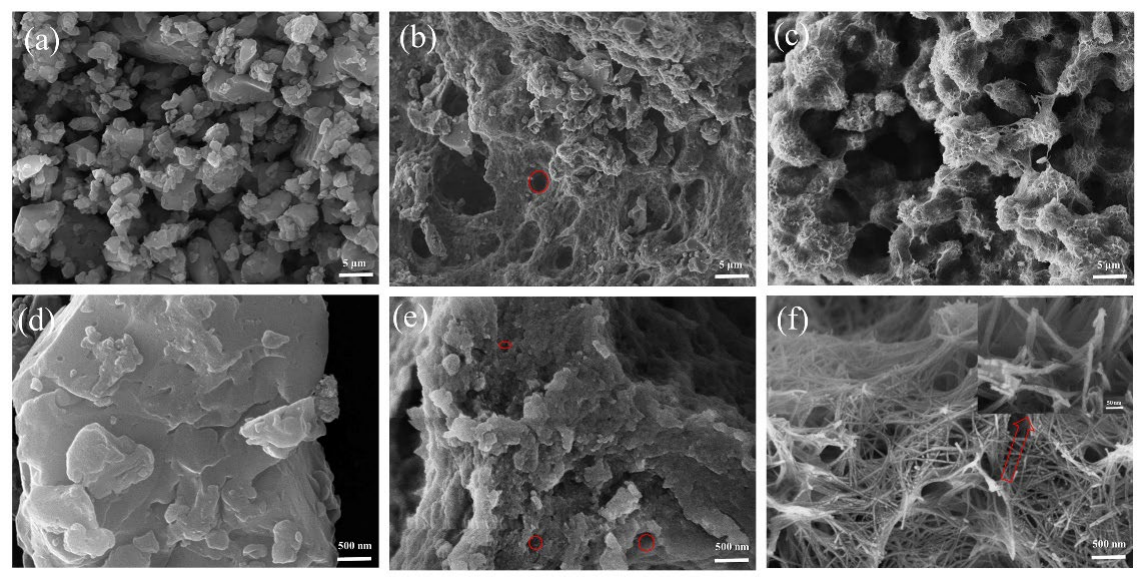
SEM pictures: (**a**) TL with 5 kx; (**b**) AL with 5 kx; (**c**) AL-TiNTs with 5 kx; (**d**) TL with 50 kx; (**e**) AL with 50 kx; (**f**) AL-TiNTs with 50 kx and 200 kx.

In Figure 1, the SEM images of TL, AL, and AL-TiNTs are shown. When the magnification was 5 kx, the pure TL particles were aggregated into a blocky structure (Figure 1a), the modified AL exhibited a more compact structure with some pores formed by depressions on the surface (Figure 1b), and the AL-TiNTs presented a fluffy structure with an irregular spherical shape formed by the surface projections and more pores (Figure 1c). When the magnification reached 50 kx, the TL surface was smooth with fewer pores (Figure 1d), while the AL surface was rough and presented a few pores (Figure 1e). On the one hand, this could be due to the modified amino grafting on the pure TL, which changed its surface structure [32]; on the other hand, the washing and freeze-drying process might leave pores [30]. In particular, the AL-TiNTs presented a randomly entangled nanotube-like structure, along with a nanotube cross-shaped porous network structure (Figure 1f), which was related to the Ti–O–Ti bond breaking and the formation of sodium titanate during the hydrothermal process [33]. Results indicated that the hydrothermal and modification synthesis of AL-TiNTs not only improved the disadvantages of lignin which was prone to agglomeration but also formed the nanotubular structure, which greatly improved the specific surface area of AL-TiNTs and thus promoted the adsorption and photocatalytic reduction properties of AL-TiNTs [34].

#### 2.1.2. EDS

**Figure 2:**
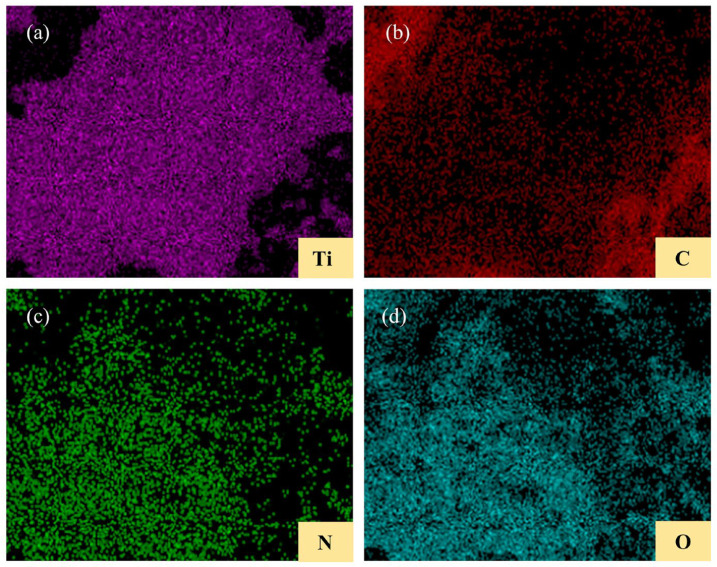
EDS mapping of AL-TiNTs: (**a**) Ti; (**b**) C; (**c**) N; and (**d**) O.

The EDS mapping of the AL-TiNTs is presented in Figure 2. The presence of the element N in Figure 2c was evidence of the presence of the amino group. The high degree of overlap between the Ti (Figure 2a) and O (Figure 2d) elements indicated the possible presence of Ti-O bonds in the AL-TiNTs, and it was assumed that the synthesis of Na_2_Ti_3_O_7_ took place. Meanwhile, the dense distribution of Ti (Figure 2a), C (Figure 2b), and N (Figure 2c) elements proved the involvement of AL in the dispersion of titanate nanotubes, further proving the successful synthesis of AL-TiNTs.

#### 2.1.3. BET

**Figure 3:**
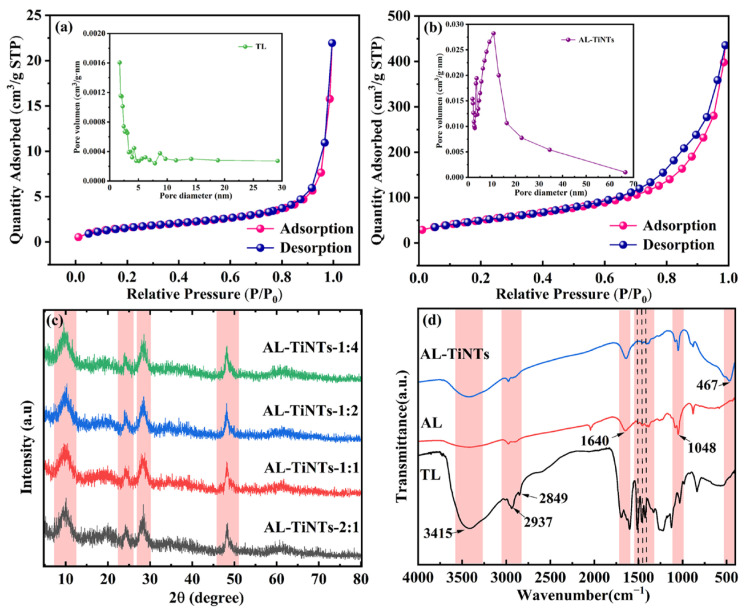
Adsorption–desorption isotherms (**a**) TL and (**b**) AL-TiNTs; (**c**) XRD patterns of the AL-TiNTs; (**d**) FT-IR spectra of AL-TiNTs, AL, and TL.

Figure 3a presents a type II isotherm of a very small H3 hysteresis loop, which was in accordance with the previously reported conclusion that lignin is predominantly a microporous structure and has less content of mesoporous structure [35]. Compared to the TL, although AL presented a relatively large H3 hysteresis loop (Figure S1), it was still a type II isotherm, suggesting that the amination modification was not significant in affecting the lignin pore structure and that the microporous structure was still predominant in AL. Conversely, Figure 3b presents the typical appearance of type IV isotherms and H3 hysteresis loops, showing their mesoporous structures with diameters of 2–50 nm [36]. The PSD curves calculated by the BJH method are depicted in the insets of Figure 3a,b. Compared to Figure 3a, the PSD curves in Figure 3b exhibit dramatic peaks, and the pore diameter at 3–4 nm corresponds to the pores contained within the AL-TiNT tubes [5]. The 9–12 nm corresponds to spaces between the aggregated tubes of AL-TiNTs, which was in accordance with the SEM characterization results [37]. The BET results for TL, AL, and AL-TiNTs are presented in Table S1. Compared to TL (5.9037 m^2^/g) and AL (6.7762 m^2^/g), the AL-TiNTs offered a larger specific surface area (188.51 m^2^/g), owing to the special tubular structure and resulting in a higher adsorption capacity [16].

#### 2.1.4. XRD

The composites prepared with different AL to nano-TiO_2_ ratios displayed similar XRD patterns (Figure 3c). The AL-TiNTs synthesized in different ratios presented characteristic peaks with diffraction angles of 48°, 28°, and 24.5°, which were the characteristic peaks of titanate nanotubes and belonged to the (020), (211), and (110) planes of TiNTs, respectively [38]. The characteristic peak at 10° corresponded to one of the (001) crystal planes of Na_2_Ti_3_O_7_ [39], and the peaks at 48°, 28°, and 24.5° indicated that the composite contains sodium titanate compounds and hydrogen titanate compounds [39]. The broad peak at 10° corresponded to a layer spacing of approximately 0.7–0.85 nm between layers of titanate [40]. In addition, the lack of anatase diffraction peaks demonstrated that all of the TiO_2_ was converted to titanate nanotubes [41].

#### 2.1.5. FT-IR

It was obvious that the absorption peaks of modified lignin were weakened, which was because of the involvement of the O–H bond during the Mannich reaction, resulting in the reduction of the O–H group (Figure 3d). However, following the synthesis of the AL-TiNTs, the increased absorption peak at 3415 cm^−1^ suggested the formation of a new O–H group, which could be the stretching vibration of O–H in the Ti–OH bond on the nanotubes [2]. High peaks at 2849 and 2937 cm^−1^ occurred due to the aromatic methoxy C–H vibration. Aromatic skeletal vibrational peaks at 1422, 1461, 1511, and 1600 cm^−1^ were found in TL, AL, and AL-TiNT fractions, which were typical peaks of lignin [42,43]. The C–N vibrational peak at 1048 cm^−1^ and the N–H in-plane bending vibrational peak at 1640 cm^−1^ were found in the AL and AL-TiNTs, which indicated the successful introduction of amino groups on lignin [44]. Meanwhile, the O–Ti and Ti–O–Ti bond at 467 cm^−1^ was found in AL-TiNTs [45], indicating the successful synthesis of sodium titanate Na_2_Ti_3_O_7_, which was in accordance with the SEM and XRD analyses [46].

### 2.2. Adsorption

#### 2.2.1. Mass Ratios of AL/Nano-TiO_2_

**Figure 4:**
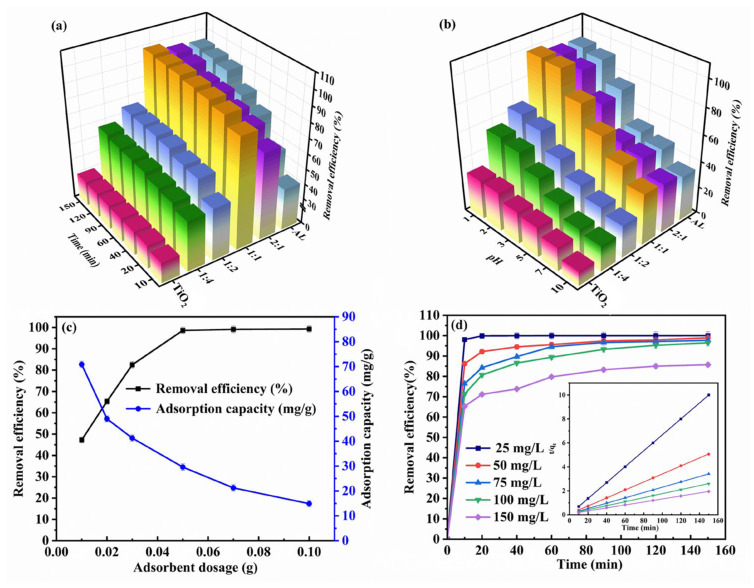
Adsorption performance of AL-TiNTs: (**a**) the mass ratio of AL/nano-TiO_2_; (**b**) pH; (**c**) AL-TiNT dosage; and (**d**) initial concentration of Cr (VI) and kinetic analysis (AL-TiNT dosage: 0.05 g; pH = 2; temperature: 25 °C).

The AL/nano-TiO_2_ mass ratio played a remarkable influence on Cr(VI) removal when the AL/nano-TiO_2_ ratios were set as 0.25, 0.5, 1, and 2. Pure AL and nano-TiO_2_ were also considered (Figure 4a). When the AL/nano-TiO_2_ mass ratio was 0.25, only 78.14% of Cr (VI) was removed. As the AL/nano-TiO_2_ mass ratio improved, the content of amino functional groups gradually increased, more adsorption active sites were exposed, and the removal efficiency increased gradually. When the mass ratio of AL/nano-TiO_2_ was 1:1, the removal efficiency could reach 99.80%, which was found to be the optimum mass ratio of AL/nano-TiO_2_.

#### 2.2.2. pH

Cr(VI) existed in three main forms in aqueous solution, mainly in CrO_4_^2−^ at pH > 6.0 and in H_2_CrO_4_ and HCrO_4_^−^ at pH < 6.0 [47]. It was found that the acidic environments were beneficial in improving the AL-TiNT adsorption capacity (Figure 4b). AL-TiNTs showed a clear downward trend of the Cr(VI) removal efficiency as the pH increased. When the solution became neutral, the removal efficiency increased slightly. Results indicated that under strongly acidic conditions, the AL-TiNTs exhibited better adsorption performance and could remove 99.80% of Cr(VI) at pH 2. The reactions during adsorption were as follows [48]:

(1) $$-NH_{3}^{+}+HCrO_{4}^{-}⇌-NH_{3}^{+}HCrO_{4}^{-}$$

(2) $$2-NH_{3}^{+}+CrO_{4}^{2-}⇌{\left(-NH_{3}^{+}\right)}_{2}CrO_{4}^{2-}$$

(3) $$2-NH_{3}^{+}+Cr_{2}O_{7}^{2-}⇌{\left(-NH_{3}^{+}\right)}_{2}Cr_{2}O_{7}^{2-}$$

#### 2.2.3. AL-TiNT Dosage

Figure 4c exhibits the effect of the AL-TiNT dosage (0.01, 0.02, 0.03, 0.05, 0.07, and 0.10 g) on the adsorption performance. When the adding amount of AL-TiNTs increased, the Cr(VI) removal efficiency gradually increased, while the AL-TiNT adsorption capacity decreased. There was a slope of the removal efficiency curve that tended to slow down between 0.02 and 0.10 g and a 100% removal of Cr(VI) by AL-TiNTs at 0.10 g. In contrast, the adsorption capacity of AL-TiNTs at the lower dosing of 0.01 g reached 70.87 mg/g, which was higher than the adsorption capacity of 0.1 g AL-TiNTs. The AL-TiNT adsorption capacity decreased sharply with increasing the dosage of AL-TiNTs from 0.01 to 0.03 g. This could be explained as follows: as the amount of the AL-TiNTs increased, the AL-TiNTs were exposed to more adsorption sites, resulting in more Cr(VI) being adsorbed on AL-TiNTs [49]. Therefore, the optimum dosage of AL-TiNTs was 0.05 g based on the removal efficiency and cost.

#### 2.2.4. Temperature

The highest Cr(VI) removal efficiency was found to be 100% at 35 °C, while it was only 96.51% at 20 °C (Figure S2). The performance of AL-TiNTs in removing Cr(VI) decreased with decreasing temperatures, which may be due to the decreased vibrational frequency of Cr(VI) at lower temperature [50].

#### 2.2.5. Cr(VI) Concentration

Figure 4d presents the effect of the initial Cr(VI) concentration (*C*_0_). As the results indicated, with the increase in *C*_0_, the removal efficiency declined. The equilibrium removal efficiency of AL-TiNTs for Cr(VI) was 85.72% at the *C*_0_ of 150 mg/L, which was comparatively lower. However, the AL-TiNT adsorption capacity was relatively higher reaching 77.15 mg/g. This could be explained by that when the dosage of AL-TiNTs was constant, the total number of active adsorption sites was also limited. At the lower Cr(VI) concentrations, the AL-TiNTs displayed a relatively greater number of adsorption sites where Cr(VI) could be adsorbed more efficiently. Adsorption sites of AL-TiNTs were continuously occupied with the increasing amount of pollutant, and thus, the excess amount of Cr(VI) could not be adsorbed.

### 2.3. Photodegradation Study

**Figure 5:**
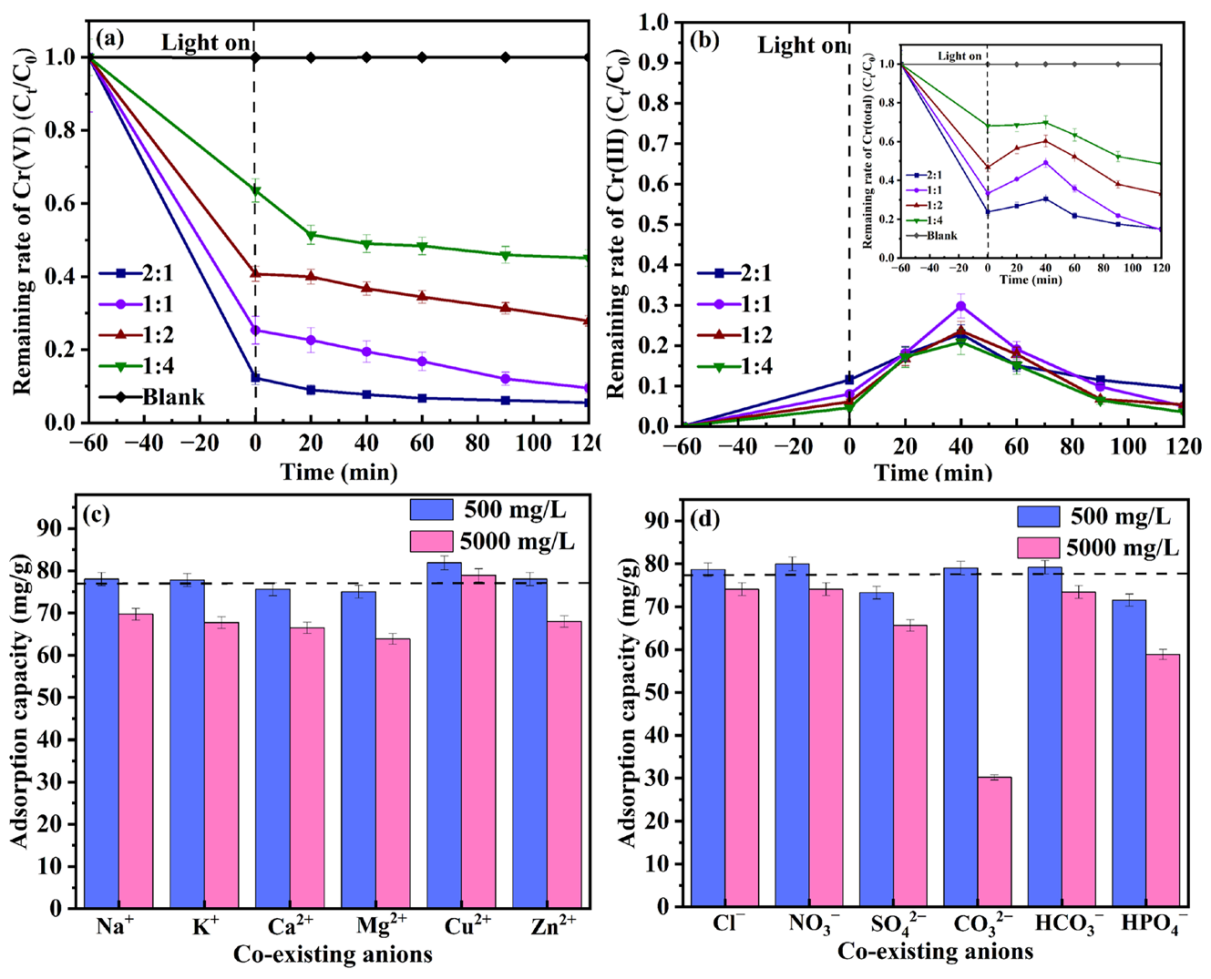
The effect of photocatalysis and co-existing anions on the performance of AL-TiNTs: (**a**) variation of Cr(VI); (**b**) variation of Cr(III) and total Cr; (**c**) cations; and (**d**) anions of different ionic strengths (AL-TiNTs: 0.05 g; pH = 2; temperature: 25 °C; xenon lamp current: 8.5 A).

The photodegradation of the AL-TiNTs was investigated under light (Figure 5a,b). At low Cr(VI) concentration (50 mg/L), the AL-TiNTs were able to adsorb almost 100% of Cr(VI). Therefore, to study the reductive efficiency of AL-TiNTs under visible light, Cr(VI) concentration with 150 mg/L was used. The removal efficiency of Cr(VI) by the AL-TiNTs of AL/nano-TiO_2_ ratios of 1:1 and 2:1 could reach 90.48% and 94.48% after the adsorption and photocatalytic reaction, respectively (Figure 5a). However, the AL-TiNT materials with the AL/nano-TiO_2_ mass ratio of 1:1 achieved a conversion of Cr(III) of up to 29.76% at 40 min under light exposure (Figure 5b). Therefore, the reduction of Cr(VI) provided more evidence of the better performance of AL-TiNTs with the AL/nano-TiO_2_ ratio of 1:1. The reason for the existence of low amounts of Cr(III) before the photocatalytic reaction was that under strongly acidic conditions, a high concentration of Cr(VI) could react with some hydrochloric acid to produce low amounts of Cr(III) (Figure 5b). After the photocatalysis, the Cr(III) concentration gradually increased. The Cr(III) in solution might have entered the titanate nanotubes and limited the complexation of electrons and holes, increasing the AL-TiNT photocatalytic activity and leading to a higher rate of Cr(III) production [35]. At 40 min, the production rate of Cr(III) decreased because of the photocatalysis, which could result from the gradual photocatalysis equilibrium at this time, and more Cr(III) could be adsorbed by the AL-TiNTs [51]. Finally, the removal amount of AL-TiNTs for total Cr was 90.00 mg/g. The reduction of AL-TiNTs for Cr(VI) following photoelectron production is displayed in Text S1 [52].

The effects of different light intensities on AL-TiNTs were investigated by varying the current of the xenon lamp light source to regulate the light intensity (Figures S3 and S4). When the operating current was 5 A, the photocatalytic performance of different AL/nano-TiO_2_ mass ratios of AL-TiNTs was poor, among which the photocatalytic reduction of AL-TiNTs with an AL/nano-TiO_2_ mass ratio of 2:1 was the worst, which was only 5.54% (Figure S4a). When the operating current was adjusted to 12 A, the photocatalytic effect of AL-TiNTs was significantly improved, and the Cr(VI) reduction efficiency of AL-TiNTs with the AL/nano-TiO_2_ mass ratio of 1:4 could reach up to 23.53%, which was twice as much as that of its Cr(VI) reduction efficiency when the current of the xenon lamp source was 5 A (Figure S4b). It could be seen that light intensity was crucial for the photocatalytic activity of AL-TiNTs.

### 2.4. Co-Existing Anions

When the co-existing ion concentrations were 5 and 50 mg/L, the influence of co-existing ions on Cr(VI) removal can be neglected (Figure S5), indicating that AL-TiNTs were highly resistant to complex solutions. It was found that Cu^2+^ and Zn^2+^ had a positive impact on the Cr(VI) removal by AL-TiNTs when each of the co-existing cation concentrations was 500 mg/L (Figure 5c). However, the adsorption performance of AL-TiNTs was slightly inhibited by the high concentration of Ca^2+^ and Mg^2+^ (5000 mg/L). On the one hand, this may be due to the high ion concentration which shielded the electrostatic attraction. On the other hand, the high concentration of the co-existing cation significantly hindered the movement of Cr(VI) to the AL-TiNTs [53]. The six co-existing anions exhibited a slight influence on the AL-TiNT adsorption performance. However, at the high co-existing anion concentration of 5000 mg/L, the six anions indicated different degrees of inhibition of the removal capacity of AL-TiNTs (Figure 5d). This could be explained by a reduction in the AL-TiNT active sites due to the occupation by an excess amount of anions. In addition, CO_3_^2−^ inhibited Cr(VI) removal by AL-TiNTs, probably because the addition of CO_3_^2−^ could increase the pH of the solution, which was not conducive to the protonation of amino groups on the AL-TiNTs, and the electrostatic attraction was not liable to occur [29]. The inhibitory effect of SO_4_^2−^ may be attributed to the nature of SO_4_^2−^ with its greater charge and higher radius ratio (z/r) and its stronger interaction with the adsorption site [54].

### 2.5. Theoretical Study

#### 2.5.1. Adsorption Kinetics

**Figure 6:**
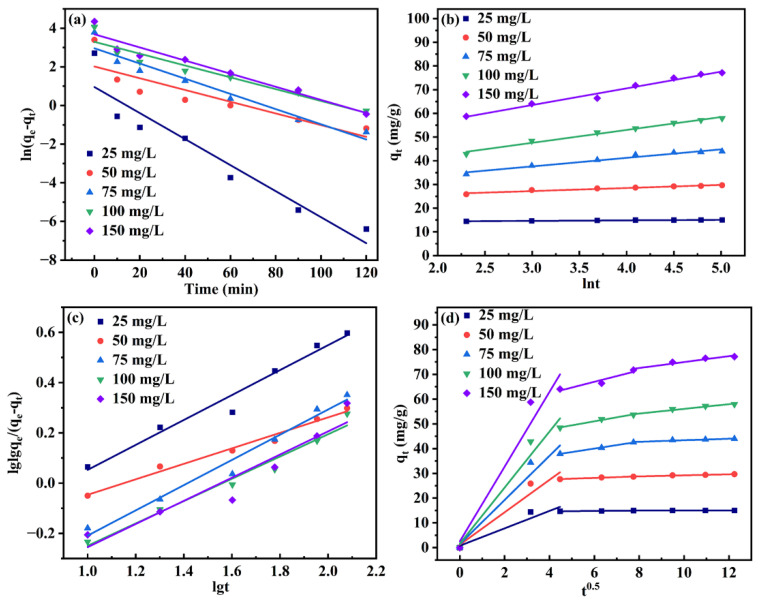
(**a**) Pseudo-first-order kinetic; (**b**) Elovich; (**c**) Bingham; and (**d**) intraparticle diffusion models (AL-TiNTs: 0.05 g; pH = 2; temperature: 25 °C).

The kinetic studies were performed at different Cr(VI) concentrations at 298 K (Figure 4d and Figure 6, Text S2). The pseudo-second-order kinetic model (Figure 4d) and the largest *R*^2^ values (0.9991–0.9999) for the kinetic model (Table S2) suggested that the secondary kinetic model was highly relevant to the Cr(VI) adsorption process. Compared to other kinetic models (Figure 6a–d), the theoretical equilibrium adsorption capacities of the proposed secondary kinetic model showed a high degree of similarity to the actual equilibrium adsorption capacities, demonstrating that the kinetic model exactly described the adsorption process of the AL-TiNTs [55]. It could be concluded that processes such as electron transfer and the sharing of active sites between Cr(VI) and the AL-TiNTs occurred at the microscopic level with chemisorption playing a crucial role. Both the second and third curves did not pass through the origin of the coordinates in the fitted curves of the intraparticle diffusion model (Figure 6d), and the fit was poor. This indicated that intraparticle diffusion would not be the single limiting factor and would not be as dominant as chemical interactions.

#### 2.5.2. Adsorption Isotherms

Adsorption was analyzed using four different isotherm models such as Langmuir (Figure S6a), Freundlich (Figure S6b), Temkin (Figure S6c), and D-R isotherm models (Figure S6d), and the details are presented in Text S3. The highest *R*^2^ value (0.9912–0.9972) for the Langmuir model in Table 1 suggested that the Langmuir model described the adsorption processes involved in this study to a considerable extent, suggesting a consistent adsorption process for single-molecular-layer adsorption [35]. At different temperatures, the *R_L_* < 1 indicated that AL-TiNTs readily adsorb Cr(VI) [27]. The *R_L_* value diminished as the concentration increased, indicating that the adsorption process of AL-TiNTs was irreversible as Cr(VI) concentration increased, and there are intense interactions between the AL-TiNTs and the adsorbate molecules Cr(VI) [56]. In the Freundlich isotherm fit results, 0 < 1/n < 1 indicated that the adsorption process involved in this study was easy to carry out, which led to the same conclusion as that obtained from the Langmuir isotherm fit [57]. Furthermore, in the D-R model, the high *E* value (>8 kJ/mol) indicated that there was a predominantly chemisorption process for the AL-TiNTs.

#### 2.5.3. Thermodynamics

A thermodynamic approach to reveal the orientation and complexity of the reaction process is shown in Figure S7 and Text S4. The consequences are given in Table S3. A negative value for Δ*G*_0_ indicated that the reaction was spontaneous. It was confirmed by Δ*H*_0_ > 0 that the AL-TiNT adsorption process was heat-absorbing. Δ*S*_0_ > 0 implied an improvement in the stoichiometry of the AL-TiNT/solution interface [50].

### 2.6. Selective Adsorption

**Figure 7:**
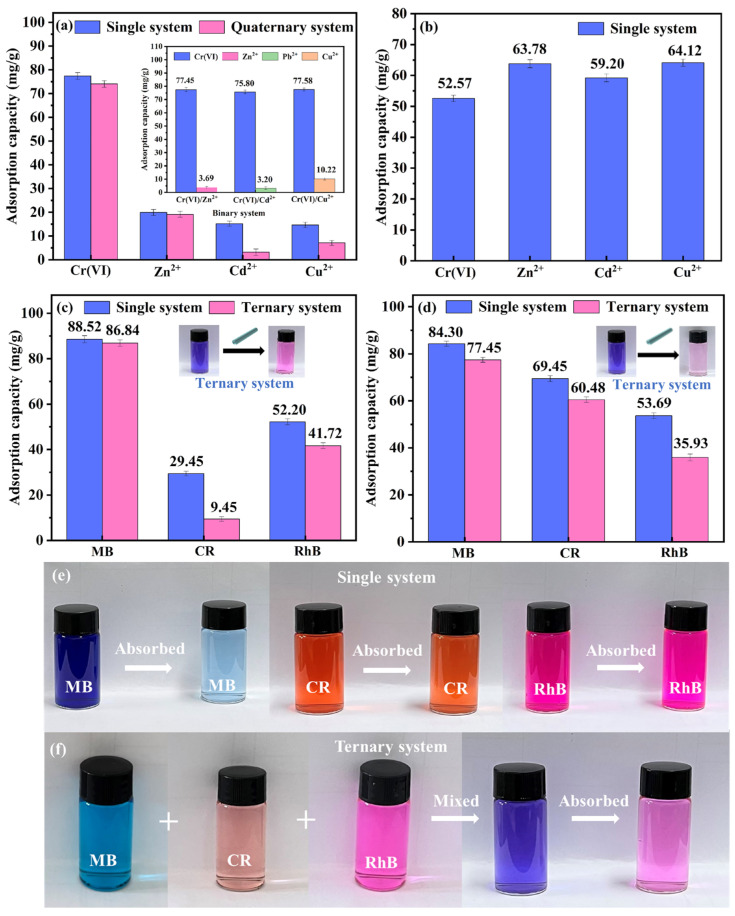
Adsorption of AL-TiNTs on heavy metal in single, binary, and quaternary systems (**a**) pH = 2 and (**b**) pH = 7; adsorption of AL-TiNTs on dyes in single and ternary systems (**c**) pH = 7 and (**d**) pH = 5.5; color change of dyes before and after adsorption by AL-TiNT (**e**) single system and (**f**) ternary system (AL-TiNTs: 0.05 g; temperature: 25 °C).

The selective adsorption performance of AL-TiNTs on heavy metals was investigated (Figure 7a). The methods for the analysis of Zn^2+^, Cd^2+^, and Cu^2+^ are presented in Text S5. Under acidic conditions (pH = 2), AL-TiNTs exhibited excellent adsorption performance for Cr(VI), while poorer adsorption performance for Zn^2+^, Cd^2+^, and Cu^2+^ was observed. These results were also found in the binary and quaternary systems. The reason may be that Cr(VI) appeared in the solution as the negatively charged anions (Cr_2_O_7_^2−^, CrO_4_^2−^, and HCrO_4_^−^), which were more easily adsorbed to the protonated amino groups on AL-TiNTs by the electrostatic attraction. It could be demonstrated that under acidic conditions, titanate nanotubes (TiNTs) were positively charged, while Zn^2+^, Cd^2+^, and Cu^2+^ were also positively charged metal ions, which could be electrostatically repelled by AL-TiNTs [58]. Ultimately, these heavy metal ions could only weakly physically adsorb through the pores of the AL-TiNTs. The adsorption performance of AL-TiNTs on Zn^2+^, Cd^2+^, and Cu^2+^ under neutral conditions without pH adjustment is displayed in Figure 7b. Interestingly, a significant improvement in the adsorption capacities of Zn^2+^ (63.78 mg/g), Cd^2+^ (59.20 mg/g), and Cu^2+^ (66.35 mg/g) by AL-TiNTs was observed. It revealed that under acidic conditions, AL-TiNTs performed exceptional selective adsorption of Cr(VI), and under neutral conditions, AL-TiNTs performed broad-spectrum adsorption of heavy metals, which was beneficial to the wide application of AL-TiNTs in heavy-metal-containing wastewater treatment.

AL-TiNTs were added to the solutions containing methylene blue (MB), Congo red (CR), and rhodamine B (RhB). The selective adsorption performance of AL-TiNTs on dyes was investigated in the mixed ternary system at different pH conditions, respectively (Figure 7c,d). The methods for the testing of MB, CR, and RhB are described in Table S4. Results revealed that under neutral conditions, the adsorption performance of AL-TiNTs on dyes was in the order of MB > RhB > CR. Considering that the discoloration range of CR was 3.5–5.2, the adsorption of AL-TiNTs for CR was further investigated by adjusting the pH to 5.5 (Figure 7d). There was a remarkable change in the adsorption performance of AL-TiNTs for CR (69.45 mg/g). However, in the mixed ternary system, AL-TiNTs still prefer to adsorb MB. Under neutral conditions without pH adjustment, MB exhibited the most obvious color change from dark blue to light blue, while CR and RhB showed very little color change in the single system, (Figure 7e). In the mixed ternary system, the color changed from blue-purple to light pink because of the selective adsorption of MB (86.86 mg/g) by the AL-TiNTs, and only 41.72 and 9.45 mg/g were found for RhB and CR, respectively (Figure 7f). The excellent selective adsorption ability of AL-TiNTs for MB may be explained by the π-π stacking and electrostatic interactions between MB molecules and the aromatic rings on AL-TiNTs [59,60]. On the contrary, the spatial site resistance caused by the long side chains of the RhB molecule would inhibit its π-π stacking and electrostatic attraction with the AL-TiNTs, making it difficult for RhB to be removed by AL-TiNTs. The low AL-TiNT adsorption capacity for CR was attributed to the fact that both TiNTs and CR were negatively charged under neutral conditions, which may produce a strong electrostatic repulsion [61]. The results indicated that AL-TiNTs exhibited strong selective adsorption of MB in a wide pH range, which was beneficial for the application of AL-TiNTs in dye-containing wastewater treatment.

### 2.7. Mechanism Study

**Figure 8:**
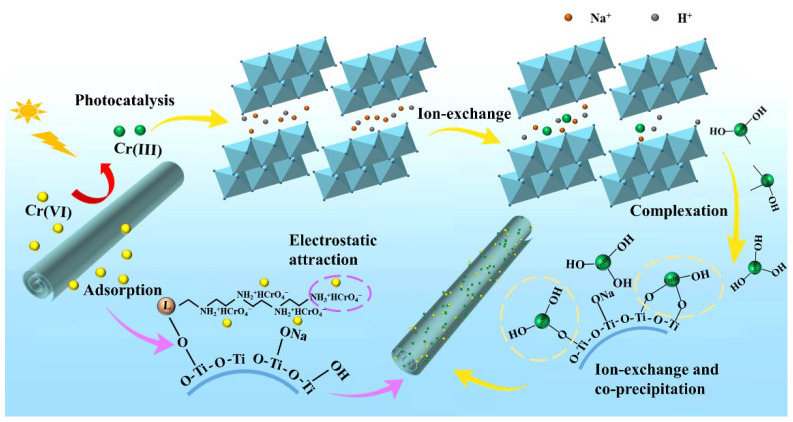
The mechanism of Cr(VI) removal by AL-TiNTs.

Figure 8 explains the removal mechanism of AL-TiNTs for Cr(VI), which involved the following processes:

(1). The amino group in AL grafted onto titanate nanotubes was protonated and adsorbed Cr(VI) by electrostatic attraction under acidic conditions;
(2). Under visible light, the AL-TiNTs generated electrons, and the Cr(VI) adsorbed on the AL-TiNTs was reduced to Cr(III);
(3). The positively charged Cr(III) reached the electronegative surface of the titanate nanotubes by electrostatic attraction and was eventually absorbed by the titanate nanotubes through ion exchange with Na^+^ and H^+^ between the nanotube layers or through complexation and co-precipitation [4].

### 2.8. Regenerative and Applicative Study

**Figure 9:**
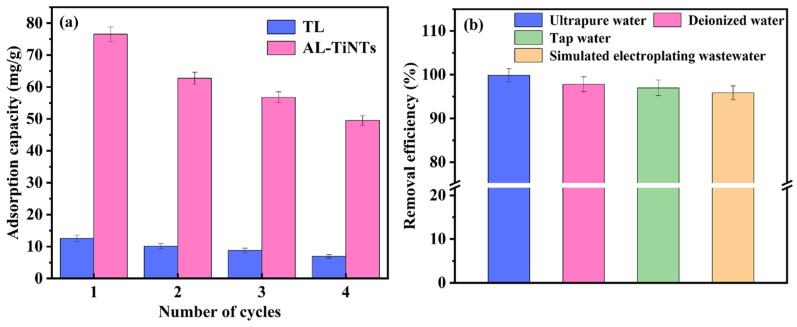
(**a**) The reusability of AL-TiNTs; (**b**) the applicability of AL-TiNTs in different water resources. (AL-TiNTs: 0.05 g; pH = 2; temperature: 25 °C.).

The durability and stability of the material are important indicators for assessing the performance of the AL-TiNT materials, as well as for evaluating their application prospects. It was appropriate to use NaOH solution as the desorption reagent for the reasons presented in Text S6. The effect of desorption solution pH on the desorption efficiency of Cr(VI) was explored (Figure S8). The result indicated that the desorption efficiency was essentially stable at 81.32% when the pH value was greater than 10. Continuing to increase the pH value, the desorption efficiency was hardly improved. The adsorption capacity of TL and AL-TiNTs diminished with the number of applications (Figure 9a). On the one hand, the structure of the AL-TiNTs may have changed after several adsorption–desorption cycles. On the other hand, the re-obtained AL-TiNTs after the adsorption of Cr(VI) (AL-TiNTs-Cr) are more stable compared to the original AL-TiNTs, and many active sites may be blocked. In addition, in the first-time application, the adsorption capacity for Cr(VI) was seven to eight times higher by AL-TiNTs than that by TL. After four cycles, the adsorption capacity of AL-TiNTs was 49.50 mg/g, which was much higher than the adsorption capacity of amino-modified titanate nanotubes TNTs-RNH_2_ after three cycles (23.9 mg/g) [37]. Under the condition of the adsorbent undergoing alternating acidic and basic conditions, the AL-TiNTs still retained excellent stability.

A comparison of the adsorption capacity of AL-TiNTs and other adsorbents for Cr(VI) is presented in Table 2. The adsorption capacity of AL-TiNTs after four cycles was still higher than that of the first-used iron oxide-coated cellulose/hydrotalcite (Fe_3_O_4_@CelHT) (29.00 mg/g) [62], multi-wall carbon nanotube (MWCNT) (11.41 mg/g) [63], mesoporous carbon nitride (MCN) (48.31 mg/g) [64], and fox nutshell activated carbon (FNAC) (46.21 mg/g) [65].

The applicability of AL-TiNTs in different water sources was further investigated (Figure 9b), and the detailed parameters of the simulated electroplating wastewater are described in Table S5. After 2.5 h of reaction, the removal efficiencies obtained by AL-TiNTs for Cr(VI) were 97.81%, 96.97%, and 95.86% in the deionized water, tap water, and simulated electroplating wastewater, respectively. Although the removal efficiencies were relatively lower compared to those in ultra-pure water (99.80%), they were all above 95%, indicating the potential of AL-TiNTs for practical applications.

## 3. Materials and Methods

### 3.1. Materials and Chemicals

The TL used was the same as we mentioned before [53]. TiO_2_ (99.8%, 40 nm), potassium dichromate (K_2_Cr_2_O_7_), formaldehyde (CH_2_O), potassium ferricyanide (K_3_[Fe(CN)_6_]), triethylenetetramine (TETA), acetone (C_3_H_6_O), and phosphoric acid (H_3_PO_4_) were obtained from Damao Chemical Reagent Co., Ltd. (Tianjin, China). Further material information is provided in Text S7.

### 3.2. Preparation of AL-TiNTs

#### 3.2.1. Amination of TL

Amine modification of TL by Mannich reaction [2]: First, 5 g of TL was dissolved in 0.4 mM sodium hydroxide (NaOH) solution (30 mL). Then, 4 mL of TETA and 3.6 mL of CH_2_O were added and stirred (500 rpm) for 15 min. After that, the mixture was placed in water at 75 °C for 3 h. The mixed solution was filtered and collected. Then, an excess of K_3_[Fe(CN)_6_] solution was added to the filtrate to allow the modified cationic lignin amine to precipitate out. To obtain aminated lignin (AL), the product was cleaned with ethanol and alternating ultra-pure water and freeze-dried for 24 h. The physical pictures of TL and AL samples are presented in Figure S9a,b.

#### 3.2.2. Synthesis of AL-TiNTs

A total of 1.0 g of nano-titanium dioxide (nano-TiO_2_) powder was dissolved into 10 M NaOH solution (50 mL) with magnetic stirring (500 rpm) for 30 min. After that, 1.0 g of AL was added and stirred magnetically (500 rpm) for 45 min. Then, AL and titanium dioxide nanoparticles were well dispersed in the NaOH solution. The suspension was transported to a 100 mL PTFE-lined reactor, and the reaction was performed continuously at 150 °C for 24 h [66]. Finally, we filtered the solution and washed the precipitate to pH 7. The product was freeze-dried for 24 h and named AL-TiNTs. AL-TiNTs with AL/nano-TiO_2_ mass ratios of 2:1, 1:2, and 1:4 were obtained by varying the AL and nano-TiO_2_ addition ratios, with other steps remaining the same. The physical image of the AL-TiNT sample is shown in Figure S9c.

### 3.3. Characterizations

The morphology of TL, AL, and AL-TiNTs was investigated by scanning electron microscopy (SEM) and energy-dispersive X-ray spectroscopy (EDS) (Czech TESCAN MIRA LMS). X-ray diffraction (XRD) was obtained with an Ultima IV X-ray diffractometer (Rigaku, Tokyo, Japan). The Fourier transform infrared spectrometer (FT-IR, Nicolet iS10 spectrometer) and the specific surface area (Micromeritics ASAP 2460 Version 3.01) were performed by the KBr particle technique and Brunauer–Emmett–Teller (BET) technique, respectively.

### 3.4. Experimental Design

#### 3.4.1. Adsorption Experiment

A total of 0.05 g of AL-TiNTs was added to the conical flask, which contains Cr(VI) solution (50 mg/L), and shaken well with a constant temperature shaker (150 rpm). The pH was adjusted with HCl and NaOH. Using a disposable syringe, the sample solution was aspirated at a fixed time in 2 mL and filtered. Cr(VI) was measured with UV-Vis spectrophotometer (UV6100s, MAPADA, Shanghai, China). Details of the parameters influencing the adsorption performance of AL-TiNTs are described in Text S8.

### 3.4.2. Photocatalytic Degradation Experiments

Exploring Cr(VI) adsorption by AL-TiNTs through preliminary experiments, the adsorption–desorption balance was reached at around 40 min. Therefore, 0.05 g AL-TiNTs were incorporated into Cr(VI) (150 mg/L) solution (30 mL, pH = 2) at 25 °C and agitated magnetically for 40 min; then, the 300 W xenon lamp (wavelength > 420 nm) was switched on and illuminated continuously for 120 min. Sample solutions were collected at various times during the reaction and filtered with a 0.22 μm filter membrane. The total Cr concentration was detected by inductively coupled plasma mass spectrometry (iCAP6300). The *degradation rate* was calculated by

(4) $$Degradation\;rate\;(\%)=\frac{\left(C_{0}-C_{t}\right)}{C_{0}}\times 100\%$$

where *C*_0_ (mg/L) and *C_t_* (mg/L) represent the Cr(VI) concentrations at the beginning of photocatalysis with the light source on and at time *t* (min) during the photocatalytic process, respectively.

### 3.4.3. Reusability Study

The post-use AL-TiNTs were added to 0.1 M NaOH solution and stirred to desorb Cr(VI) to investigate the *reuse efficiency* of AL-TiNTs. The calculation of Cr(VI) desorption efficiency is provided in Text S6. The *reuse efficiency* of AL-TiNTs was determined as follows:

(5) $$Reuse\;efficiency\;(\%)=\frac{q_{n}}{q_{1}}\times 100\%$$

where *q*_1_ is the amount of adsorption of AL-TiNTs for the first time, and *q_n_* is the amount adsorbed by recycling for the time *n*.

## 4. Conclusions

A novel layered nanotube material, AL-TiNT, was successfully prepared by a simple hydrothermal method. Under the synergistic effect of adsorption and photocatalysis, the AL-TiNTs provided the potential to adsorb and reduce Cr(VI) in one step. The adsorption capacity of AL-TiNTs for total Cr was 90.00 mg/g. The AL-TiNTs exhibited strong resistance to complex solutions and showed excellent selectivity for Cr(VI) in a mixed system of quaternary heavy metal ions at pH 2. In a ternary dye-mix system, the AL-TiNTs were also selective for the adsorption of MB at pH 7. In addition, AL-TiNTs displayed broad-spectrum adsorption of Zn^2+^, Cd^2+^, and Cu^2+^ in a neutral environment. The adsorption mechanisms of AL-TiNTs were mainly monolayer adsorption, electrostatic interaction, ion exchange, and complexation. AL-TiNTs performed well (95.86%) in simulated electroplating wastewater, which has the potential for practical application.

## Figures and Tables

**Table 1:** Fitting results for different sorption isotherm models.

Isotherm Model	Parameter	Temperature (K)			
		293 K	298 K	303 K	308 K
Langmuir	*q_m_* (mg/g)	88.97	89.85	88.18	86.13
	*K_L_* (L/mg)	0.24	0.43	0.61	1.12
	*R* ^2^	0.9923	0.9972	0.9948	0.9912
Freundlich	*K_F_* ((mg/g) (L/mg)^1/n^)	30.39	37.83	42.65	49.86
	1/n	0.30	0.28	0.24	0.21
	*R* ^2^	0.9843	0.9839	0.9905	0.9774
Temkin	*K_T_* (L/mol)	3.34	7.41	15.30	49.06
	*B*_1_ (J/mol)	17.66	16.63	14.82	12.80
	*R* ^2^	0.9856	0.9893	0.9891	0.9731
Dubinin–Radushkevich	*q_m_* (mol/g)	0.0042	0.0041	0.0037	0.0033
	*K_D_* (mol^2^/g^2^)	0.0029	0.0025	0.0020	0.0016
	*R* ^2^	0.9866	0.9879	0.9917	0.9804
	*E* (kJ/mol)	13.09	14.29	15.69	17.68

**Table 2:** Comparison of Cr(VI) adsorption capacity of AL-TiNTs with some reported adsorbents.

Adsorbent	*Q_m_* (mg/g)	References
AL-TiNTs	77.15	This study
TNTs-RNH_2_	69.10	[37]
Fe_3_O_4_@CelHT	29.00	[62]
MWCNT	11.41	[63]
MCN	48.31	[64]
FNAC	46.21	[65]

## Data Availability

Not applicable.

## Supplementary Materials

The following supporting information can be downloaded at https://www.mdpi.com/article/10.3390/molecules28155789/s1. References [61,67,68,69,70] are cited in Supplementary Materials. Figure S1: Adsorption-desorption isotherms and pore distribution of AL; Figure S2: Effect of temperature on the adsorption of Cr (VI) by AL-TiNTs and kinetic analysis; Figure S3: Physical diagrams of light intensity corresponding to different xenon lamp source currents: (a) 5 A; (b) 8.5 A; (c) 12 A; Figure S4: Effect of different xenon lamp light source currents on the removal of Cr(VI) by AL-TiNTs: (a) 5 A; (b) 12 A (AL-TiNTs: 0.05 g; pH = 2; temperature: 25 °C); Figure S5: The effect of co-existing anions insolution on removal of Cr(VI) by AL-TiNTs: (a) cations and (b) anions of different ionic strengths (AL-TiNTs: 0.05 g; solution pH = 2; experimental temperature: 25 °C); Figure S6: Adsorption isotherms (a) Langmuir; (b) Freundlich; (c) Temkin; and (d) D-R isotherm models (AL-TiNTs: 0.05 g; pH = 2); Figure S7: Thermodynamics; Figure S8: Effect of pH on desorption efficiency of Cr(VI); Figure S9: (a) TL; (b) AL; (c) AL-TiNTs. Table S1: Pore structures and specific surface areas of samples; Table S2: Fitting results for different sorption models; Table S3: Thermodynamic parameters for Cr(VI) removal by AL-TiNTs; Table S4: Details of MB, CR and RhB and detection wavelengths; Table S5: The content of substances in simulated wastewater; Text S1: The reduction process of Cr(VI); Text S2: Kinetics models; Text S3: Isotherm models; Text S4: Thermodynamic; Text S5: Measurement methods of Zn^2+^, Cd^2+^, and Cu^2+^; Text S6: Desorption of Cr(VI); Text S7: Materials; Text S8: Calculation equations of removal efficiency and capacity.
