# Supplementary material for: Hydrothermal Synthesis of a Technical Lignin-Based Nanotube for the Efficient and Selective Removal of Cr(VI) from Aqueous Solution
Source: Molecules. 2023 Jul 31;28(15):5789. doi: 10.3390/molecules28155789 (PMC10421463; doi:10.3390/molecules28155789)
Supplement: Supplementary file 1 [file molecules-28-05789-s001.zip › molecules-2519736-supplementary.pdf]

Supplementary Information

Hydrothermal Synthesis of a Technical Lignin-Based Nanotube for the Efficient and Selective Removal of Cr(VI) from Aqueous Solution

Qiongyao Wang [1,†], Yongchang Sun [1,*,†], Mingge Hao [1], Fangxin Yu [1] and Juanni He [2,*]

[1] Key Laboratory of Subsurface Hydrology and Ecological Effects in Arid Region, Ministry of Education, School of Water and Environment, Chang'an University, Xi'an 710054, China; wqy03218520@163.com (Q.W.); haomingge_813@163.com (M.H.); y526328876@foxmail.com (F.Y.)

[2] Huijin Technology Holding Group Corporation Limited, Xi'an 710000, China

* Correspondence: sunyongchang60@163.com (Y.S.); jnhe2007@163.com (J.H.); Tel.: +86-29-82339952 (Y.S.)

† These authors contributed equally to this work.

List of the Supplemental Texts

Text S1. The reduction process of Cr(VI).

Text S2. Kinetics models.

Text S3. Isotherm models.

Text S4. Thermodynamic.

Text S5. Measurement methods of Zn^{2+}, Cd^{2+}, and Cu^{2+}.

Text S6. Desorption of Cr(VI).

Text S7. Materials.

Text S8. Calculation equations of removal efficiency and capacity.

List of the Supplemental Figures

Figure S1. Adsorption-desorption isotherms and pore distribution of AL.

Figure S2. Effect of temperature on the adsorption of Cr (VI) by AL-TiNTs and kinetic analysis.

Figure S3. Physical diagrams of light intensity corresponding to different xenon lamp source currents: (a) 5 A; (b) 8.5 A; (c) 12 A.

Figure S4. Effect of different xenon lamp light source currents on the removal of Cr(VI) by AL-TiNTs: (a) 5 A; (b) 12 A (AL-TiNTs: 0.05 g; pH = 2; temperature: 25 °C).

Figure S5. The effect of co-existing anions insolution on removal of Cr(VI) by AL-TiNTs: (a) cations and (b) anions of different ionic strengths (AL-TiNTs dosing amount:0.05 g; solution pH = 2; experimental temperature:25 °C).

Figure S6. Adsorption isotherms (a) Langmuir; (b) Freundlich; (c) Temkin; and (d) D-R isotherm models (AL-TiNTs: 0.05 g; pH = 2).

Figure S7. Thermodynamics.

Figure S8. Effect of pH on desorption efficiency of Cr(VI).

Figure S9. (a) TL; (b) AL; (c) AL-TiNTs.

List of the Supplemental Tables

Table S1. Pore structures and specific surface areas of samples**.**

Table S2. Fitting results for different sorption models.

Table S3. Thermodynamic parameters for Cr(VI) removal by AL-TiNTs.

Table S4. Details of MB, CR and RhB and detection wavelengths.

Table S5. The content of substances in simulated wastewater.

Texts

Text S1

The reduction of Cr(VI) by AL-TiNTs following photoelectron production under UV light proceeds was carried out as follows:

$$Cr_2O_7^{2-} + 14H^+ + 6e^- \rightleftharpoons 2Cr^{3+} + 7H_2O \tag{1}$$

$$CrO_4^{2-} + 8H^+ + 3e^- \rightleftharpoons Cr^{3+} + 4H_2O \tag{2}$$

$$HCrO_4^- + 7H^+ + 3e^- \rightleftharpoons Cr^{3+} + 4H_2O \tag{3}$$

Text S2

Pseudo-first-order kinetic model (Figure 6a):

$$\ln(q_e - q_t) = \ln q_e - K_1 t \tag{4}$$

q_e and q_t (mg/g) refer to the amount adsorbed at equilibrium and at time t (min) respectively. K_1 (min^{-1}) refers to the rate constant [1].

Pseudo-second-order kinetic model(Figure 4d)：

$$\frac{t}{q_t} = \frac{1}{K_2 \times q_e^2} + \frac{1}{q_e} \times t \tag{5}$$

K_2 ($g \cdot mg^{-1} min^{-1}$) refers to the rate constant [2].

The Elovich model (Figure 6b) ：

$$q_t = \frac{1}{\beta_E}\ln(\alpha_E \beta_E) + \frac{1}{\beta_E}\ln t \tag{6}$$

α_E($mg \cdot g^{-1} min^{-1}$) refers to the initial adsorption rate constant; β_E (g·mg) is the initial desorption rate constant.

Bangham model equation (Figure 6c):

$$\lg\lg\left[\frac{q_e}{q_e-q_t}\right]=\left[\frac{K_0}{2.303}\right]+\alpha\lg t \tag{7}$$

K_0 (mL·g^{-1}L^{-1}) is the rate constant.

Intraparticle diffusion model (Figure 6d): $t^{0.5}$ is the horizontal axis and q_t is the vertical axis. If the internal diffusion of the particle is the one factor limiting the velocity in the adsorption process, then the image obtained is a straight line. If the line of internal diffusion of the particle crosses the origin, this means that the internal diffusion of the particle is the only factor[3].

Text S3

The Langmuir isotherm model (Figure S3a) ：

$$\frac{C_e}{q_e}=\frac{1}{q_m\cdot K_L}+\frac{C_e}{q_m} \tag{8}$$

$$R_L=\frac{1}{1+K_L\cdot C_0} \tag{9}$$

C_e (mg/L) refers to the concentration at adsorption equilibrium, q_e (mg/g) refers to the amount adsorbed at equilibrium, q_m (mg/g) refers to the saturation adsorption capacity according to monolayer theory, and K_L (L/mg) refers to the constant of the Langmuir isothermal adsorption equation.The value of R_L ($1 \geq R_L \geq 0$) represents the degree of adsorption [4].

Freundlich isotherm model (Figure S3b):

$$\ln q_e=\ln K_F+\frac{1}{n}\ln C_e \tag{10}$$

K_F refers to the Freundlich equation parameter, representing the adsorption capacity of the adsorbent. n refers to the factor less parameter, representing the adsorption strength of the adsorbent. When $0 < 1/n < 1$, adsorption is easy; when $1/n = 1$, the adsorption process is independent of the concentration of the adsorbent; when $1/n > 1$, there is a strong interaction

between the adsorbent molecules; when 1/n > 2, the adsorption is difficult [5].

Temkin isotherm model (Figure S3c):

$$q_e = B_1 \ln K_T + B_1 \ln C_e \tag{11}$$

B_1 (J/mol) refers to the constant associated with the heat of adsorption. K_T (L/mol) refers to Temkin's constant.

Dubinin-Radushkevich (D-R) isotherm model (Figure S3d):

$$\ln Q_e = \ln Q_m - K_D \varepsilon^2 \tag{12}$$

Q_e (mol/g) refers to the adsorption volume at equilibrium; Q_m (mol/g) refers to the saturation adsorption capacity of the adsorbent; K_D (mol^2/kJ^2) is the D-R adsorption constant; ε indicates the Polanyi potential; T (K) indicates the solution temperature; and E (kJ/mol) indicates the type of adsorption.

Text S4

The thermodynamic parameters include the standard entropy ΔS^0, the standard enthalpy ΔH^0 and the Gibbs free energy ΔG^0 with the following equations.

$$\Delta G^0 = -\mathrm{R}T \ln K_D \tag{13}$$

$$\ln K_D = -\Delta H^0 / \mathrm{R}T + \Delta S^0 / \mathrm{R} \tag{14}$$

where K_D is the adsorption equilibrium constant, which can be defined by q_e/C_e and R is the universal gas constant (8.314 $J \cdot mol^{-1} \cdot K^{-1}$). The value of ln K_D was derived by fitting ln K_D to C_e and using the intercept. The values of ΔH^0/R and ΔS^0/R were obtained by fitting ln K_D to 1/T

Text S5

Dithizone spectrophotometry for the determination of Zn^{2+} at 535 nm is based on the formation of a red chelate of zinc ions with dithizone in an acetate buffered medium at pH 4.0–5.5.

Dithizone spectrophotometry for the determination of Cd^{2+} at 518 nm is based on the formation of a red complex of cadmium ions with dithizone in a strongly alkaline solution.

Biscyclohexanone oxaldihydrazone spectrophotometric method for the determination of Cu^{2+} at 600 nm, based on the formation of a blue chelate of copper ions with biscyclohexanone oxaldihydrazone in an ammonium chloride buffered medium at pH 9.

Text S6

Amino groups in AL-TiNTs adsorbed Cr(VI) by electrostatic attraction after protonation under acidic conditions. During the desorption process, the positively charged $-NH_3^+$ could become neutral or even negative with increasing pH, thus weakening the electrostatic attraction between the adsorbent surface and the Cr(VI) anion. Therefore, alkaline NaOH solutions could be used as an adsorbent.

The absorbed Cr(VI) on AL-TiNTs was desorbed for 4 h under different pH (5–13), and 0.1 M NaOH solution was used to adjust the solution pH. The desorption efficiency D (%) was calculated by

$$D = \frac{C_d - C_e}{C_0 - C_e} \times 100\% \quad (15)$$

where C_0 (mg/L), C_e (mg/L), and C_d (mg/L) were initial concentration, adsorption equilibrium concentration, and desorption equilibrium concentration, respectively.

Text S7

TiO_2 (99.8% metals basis, 40 nm), sodium bicarbonate ($NaHCO_3$), sodium bicarbonate

(NaCl), sodium sulfate (Na_2SO_4), sodium nitrate ($NaNO_3$), potassium chloride (KCl), calcium chloride ($CaCl_2$), magnesium chloride hexahydrate ($MgCl_2 \cdot 6H_2O$), copper chloride ($CuCl_2$), zinc chloride ($ZnCl_2$), cadmium chloride ($CdCl_2$), rhodamine B ($C_{28}H_{31}ClN_2O_3$), methylene blue ($C_{16}H_{18}ClN_3S$), and Congo red ($C_{32}H_{22}N_6Na_2O_6S_2$) was purchased from Macklin Biochemical Co., Ltd (Shanghai, China). All the mentioned reagents were analytically pure.

Text S8

The calculation equations were as follows:

$$Removal\ efficiency\ (\%) = (C_0 - C_t) / C_0 \times 100\% \tag{16}$$

$$Removal\ amount\ (\mathrm{mg/g}) = (C_0 - C_t) \times V / m \tag{17}$$

where the C_0 (mg/L) is the initial Cr(VI) concentration set in this study, C_t (mg/L) is the detected Cr(VI) concentration at time t (min) after the addition of AL-TiNTs, V (L) is the solution volume, and m (g) is the quality of the AL-TiNTs injected.

The influence of different conditions for the removal of Cr(VI) by AL-TiNTs was investigated, including the reaction time (from 0 to 150 min), pH (from 1 to 10), mass ratio of AL to nano-TiO_2 (2:1, 1:1, 1:2, and 1:4), temperature (from 20 °C to 35 °C), and Cr(VI) initial concentration (from 25 mg/L to 150 mg/L).

Figures

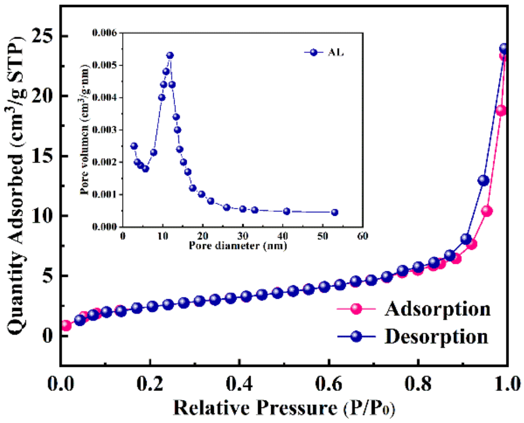

Figure S1. Adsorption-desorption isotherms and pore distribution of AL.

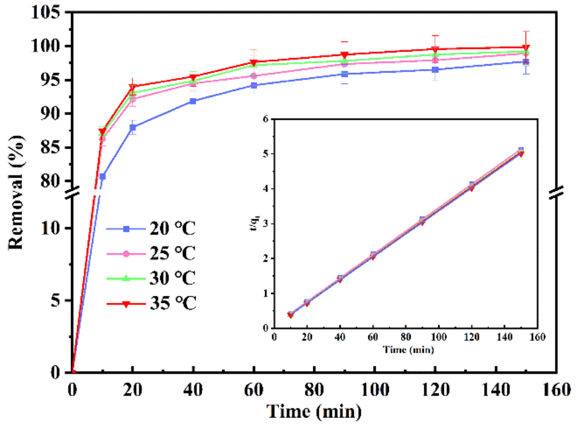

Figure S2. Effect of temperature on the adsorption of Cr (VI) by AL-TiNTs and kinetic analysis (AL-TiNTs: 0.05 g; pH = 2).

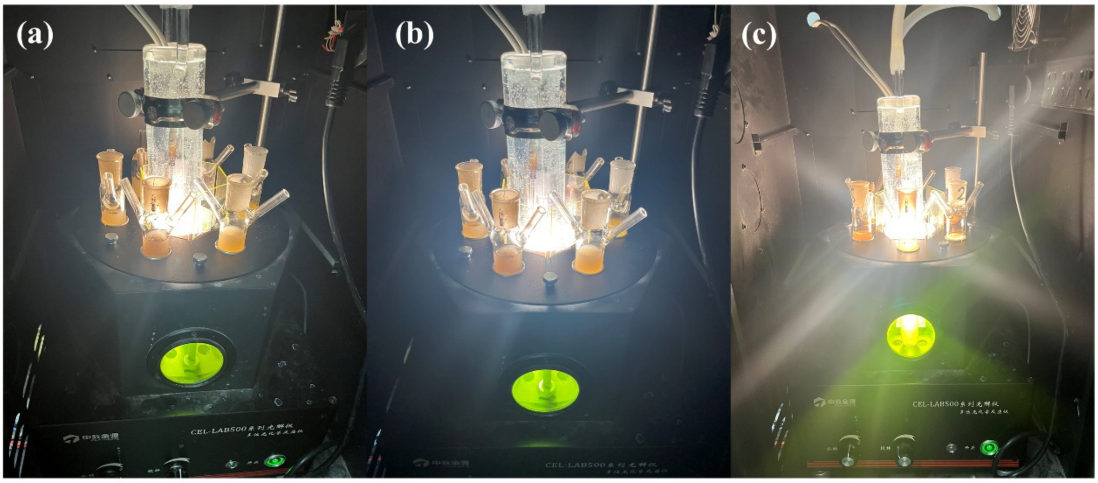

Figure S3. Physical diagrams of light intensity corresponding to different xenon lamp source currents: (a) 5 A; (b) 8.5 A; (c) 12 A.

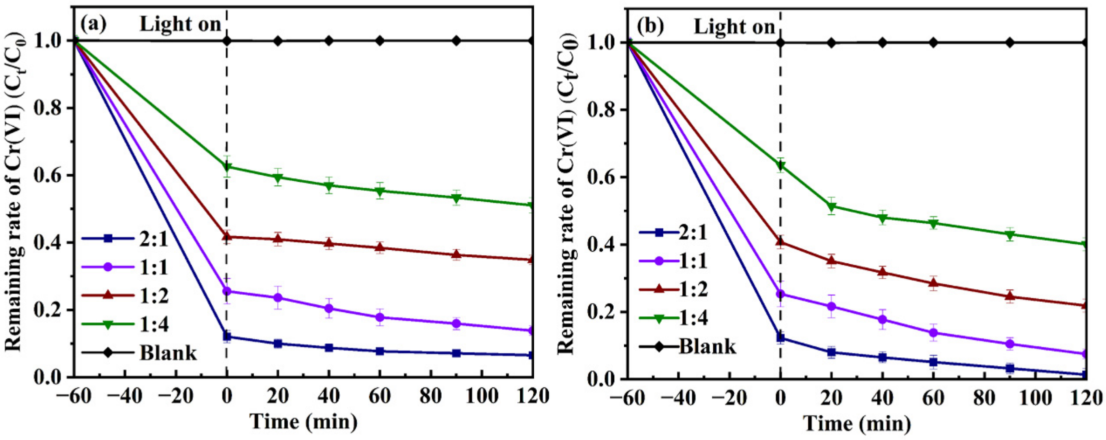

Figure S4. Effect of different xenon lamp light source currents on the removal of Cr(VI) by AL-TiNTs: (a) 5 A; (b) 12 A (AL-TiNTs: 0.05 g; pH = 2; temperature: 25 °C).

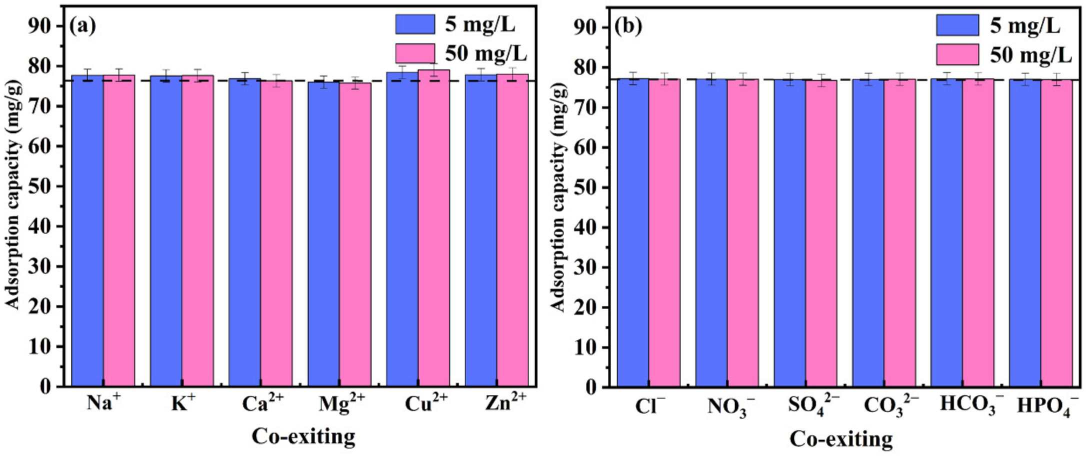

Figure S5. The effect of co-existing anions insolution on removal of Cr(VI) by AL-TiNTs: (**a**) cations and (**b**) anions of different ionic strengths (AL-TiNTs: 0.05 g; pH = 2; temperature: 25 °C).

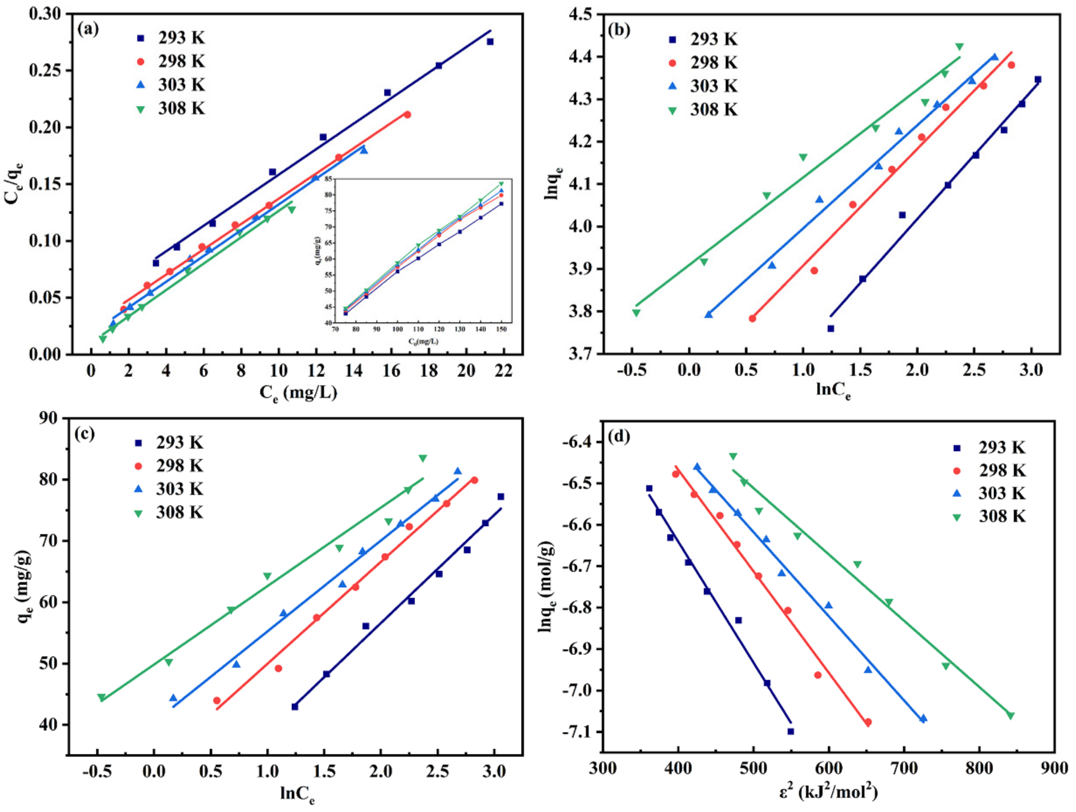

Figure S6. Adsorption isotherms (a) Langmuir; (b) Freundlich; (c) Temkin; and (d) D-R isotherm models (AL-TiNTs: 0.05 g; pH = 2).

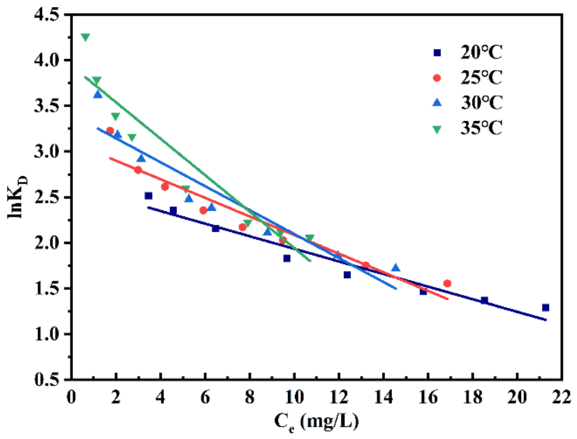

Figure S7. Thermodynamics.

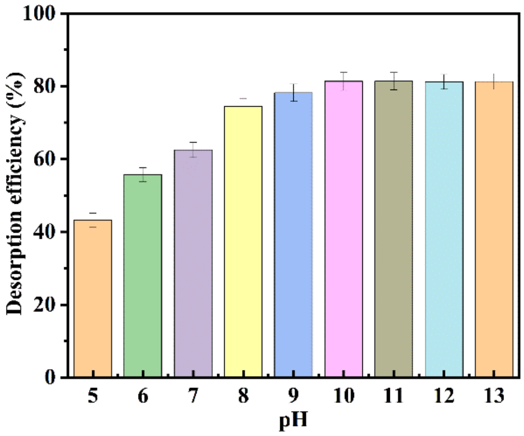

Figure S8. Effect of pH on desorption efficiency of Cr(VI).

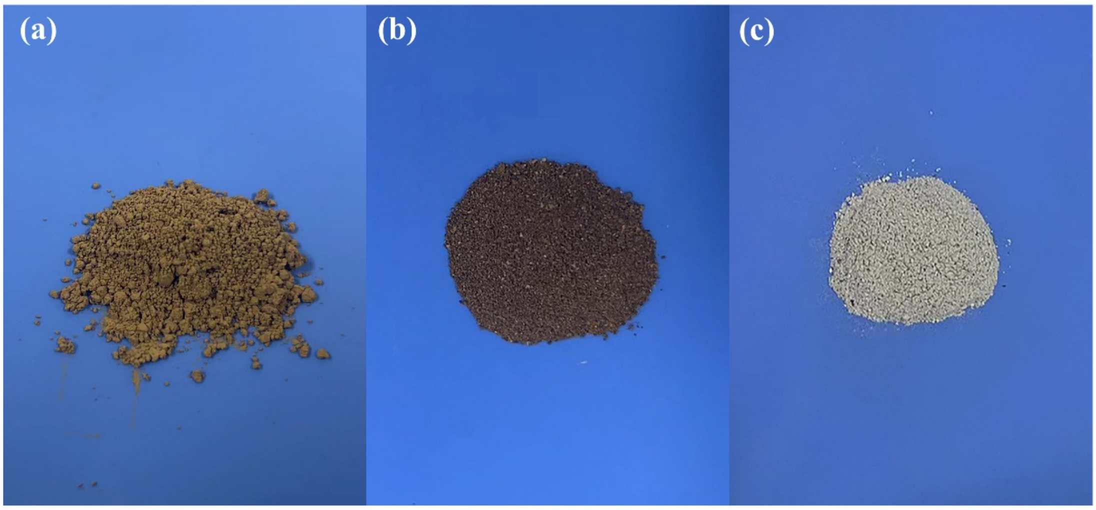

Figure S9. (a) TL; (b) AL; (c) AL-TiNTs.

Tables

Table S1. Pore structures and specific surface areas of samples

Samples	S_{BET} (m^2 g^{-1})	D_{av} (nm)[a]	V_t (cm^3 g^{-1})[b]
TL	5.9037	10.6446	0.02463
AL	6.7762	11.3954	0.06320
AL-TiNTs	188.5119	12.9449	0.42830

[a] D_{av} was the average diameter of TL, AL and AL-TiNTs.

[b] Vt represented the total volume.

Table S2. Fitting results for different sorption models.

Isotherm mode	Parameter	Initial Cr(VI) concentration (mg/L)				
		25 mg/L	50 mg/L	75 mg/L	100 mg/L	150 mg/L
Pseudo-first-order mode	Qe (mg/g)	2.59	7.51	19.23	26.91	39.79
	$K_1 \times 10^{-2}$ (min^{-1})	6.73	3.03	3.93	3.06	3.40
	R^2	0.8894	0.7373	0.9277	0.9042	0.9290
Pseudo-second-order model	Qe (mg/g)	15.06	29.95	45.05	59.52	79.74
	h (mg/(g·mg))	32.16	14.78	12.24	11.57	14.35
	$K_2 \times 10^{-2}$ [g·(mg·min)$^{-1}$]	14.18	1.65	0.60	0.33	0.23
	R^2	0.9999	0.9999	0.9998	0.9995	0.9991
Elovich model	α_E (mg/g·min)	5.8E+27	8.40E+7	6248.73	1708.49	2845.05
	β_E (g/mg)	4.68	0.77	0.28	0.19	0.15
	R^2	0.9041	0.9424	0.9570	0.9830	0.9771
Bangham model	α	0.50	0.31	0.50	0.44	0.46
	K_0 (mL/(g/L))	0.83	1.02	0.45	0.47	0.44
	R^2	0.9648	0.9790	0.9703	0.9642	0.8851
Intraparticle diffusion model	K_2 (g/mg·min$^{0.5}$)	0.090	0.32	1.40	1.61	2.31
	C_2	14.27	26.26	31.57	41.35	53.13
	R_2^2	0.9743	0.9693	0.9966	0.9722	0.8293
	K_3 (g/mg·min$^{0.5}$)	0.0051	0.21	0.31	0.95	1.20
	C_3	14.94	27.08	40.33	46.61	62.94
	R_3^2	0.7087	0.9558	0.8640	0.9509	0.9145

Table S3. Thermodynamic parameters for Cr(VI) removal by AL-TiNTs.

Temperature (K)	lnK_D	ΔG^0 (kJ·mol^{-1})	ΔH^0 (kJ·mol^{-1})	ΔS^0 (kJ·mol^{-1})
293	2.63	−6.40	63.36	0.24
298	3.10	−7.69	--	--
303	3.41	−8.58	--	--
308	3.94	−10.10	--	--

Table S4. Details of MB, CR and RhB and detection wavelengths

Dye	Abbreviation	M.W. [a]	λ_{max} [b] (nm)	Molecular formula	Molecular structure	Category
Methylene Blue	MB	319.85	664	$C_{16}H_{18}ClN_3S$	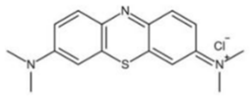	cationic
Congo Red	CR	696.66	497	$C_{32}H_{22}N_6Na_2O_6S_2$	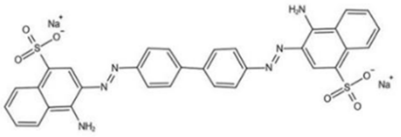	anionic
Rhodamine B	RhB	479.01	554	$C_{28}H_{31}ClN_2O_3$	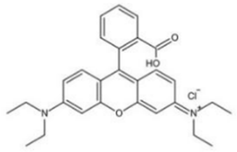	cationic

[a] Molecular Weight (g/mol)

[b] maximum absorption wavelength (nm)

Table S5. The content of substances in simulated wastewater.

Simulated electroplating wastewater			
**Substances**	**Concentration (mg/L)**	**Substances**	**Concentration (mg/L)**
Cr(VI)	50.0	Zn^{2+}	6.7
Cu^{2+}	12.4	Ca^{2+}	15.0
Ni^{2+}	5.3	Cl^{-}	53.9
Fe^{3+}	5.5	SO_4^{2-}	126.5
Al^{3+}	3.4	COD	-
pH	2	-	-

References

1. Liu, W.; Zhao, X.; Wang, T.; Fu, J.; Ni, J. Selective and irreversible adsorption of mercury(II) from aqueous solution by a flower-like titanate nanomaterial. *J. Mater. Chem. A* **2015**, *3*, 17676-17684, doi:10.1039/c5ta04521e.
2. Xiong, Z.; Zheng, H.; Hu, Y.; Hu, X.; Ding, W.; Ma, J.; Li, Y. Selective adsorption of Congo red and Cu(II) from complex wastewater by core-shell structured magnetic carbon@zeolitic imidazolate frameworks-8 nanocomposites. *Sep. Purif. Technol.* **2021**, *277*, 119053, doi:10.1016/j.seppur.2021.119053.
3. Wang, Z.; Guo, J.; Jia, J.; Liu, W.; Yao, X.; Feng, J.; Dong, S.; Sun, J. Magnetic Biochar Derived from Fenton Sludge/CMC for High-Efficiency Removal of Pb(II): Synthesis, Application, and Mechanism. *Molecules* **2023**, *28*, doi:10.3390/molecules28134983.
4. Yu, K.L.; Lee, X.J.; Ong, H.C.; Chen, W.H.; Chang, J.S.; Lin, C.S.; Show, P.L.; Ling, T.C. Adsorptive removal of cationic methylene blue and anionic Congo red dyes using wet-torrefied microalgal biochar: Equilibrium, kinetic and mechanism modeling. *Environ. Pollut.* **2021**, *272*, 115986, doi:10.1016/j.envpol.2020.115986.
5. Sun, Y.; Wang, T.; Han, C.; Lv, X.; Bai, L.; Sun, X.; Zhang, P. Facile synthesis of Fe-modified lignin-based biochar for ultra-fast adsorption of methylene blue: Selective adsorption and mechanism studies. *Bioresour. Technol.* **2022**, *344*, 126186, doi:10.1016/j.biortech.2021.126186.
